# Short foveo-disc distance in situs inversus of optic disc

**DOI:** 10.1038/s41598-020-74743-0

**Published:** 2020-10-20

**Authors:** Young In Shin, Kyoung Min Lee, Martha Kim, Sohee Oh, Seok Hwan Kim

**Affiliations:** 1 Department of Ophthalmology, Seoul National University College of Medicine, Seoul, Korea; 2 Department of Ophthalmology, Seoul National University Hospital, Seoul, Korea; 3 Department of Ophthalmology, Seoul National University Boramae Medical Center, Seoul, Korea; 4 Department of Ophthalmology, Dongguk University Ilsan Hospital, Goyang, Korea

**Keywords:** Developmental neurogenesis, Visual system

## Abstract

Situs inversus of optic disc (SIOD) is thought to be a congenital optic disc abnormality that is caused by dysversion of optic nerve insertion. SIOD, however, has many additional features that cannot be explained by abnormal optic-nerve-insertion directionality. In this study, we measured the distance between the fovea and disc in 22 eyes of 15 SIOD patients. For comparison, two control eyes were matched with each SIOD eye by age and axial length. The vertical distance between the temporal vascular arcades also was measured. The foveo-disc distance was shorter in the SIOD eyes than in the control eyes, while the inter-arcade distance did not differ. Further, we measured the circumpapillary retinal nerve fiber layer thickness, which showed nasal crowding of two humps in the SIOD eyes. This nasal crowding disappeared when we shifted the circle scan by the mean difference (465 μm) of the foveal-disc distance between the two groups. Our findings suggest that the optic disc was located closer to the fovea than it would have been normally. Thus, SIOD might reflect incomplete expansion of the posterior pole in the direction of the fovea-disc axis.

## Introduction

Situs inversus of optic disc (SIOD) is a rare optic disc abnormality characterized by emergent anomalous retinal vasculature^1,2^. The retinal vascular arcade advances nasally initially, followed by acute arching of the vessels temporally^1^. SIOD has been reported to have different peripapillary retinal nerve fiber layer (RNFL) thickness and optic nerve head (ONH) characteristics relative to normal controls^3^. Since the initial curve of the vasculature is in the reverse direction, and because this condition is commonly associated with tilted disk syndrome, SIOD is thought to be congenital and the result of dysversion of the optic disc originating from anomalous insertion of the optic stalk into the optic vesicle^3,4^.

Like other organs, every eyeball has to grow after birth. Several studies have revealed that this process is not simple linear growth^5,6^. In fact, the most significant increases in axial length occur during the first 2 years of age, after which the increases are gradual until 10 years of age. Patel et al. demonstrated this initial rapid ONH expansion period early in life in their hand-held optical coherence tomography (OCT) study^6^. During scleral expansion in this period, the retina also expands to cover the enlarged surface^7^, which leads in turn to expansion of the retinal vascular arcade and increased foveo-disc distance in the posterior polar area. If the scleral and retinal expansion is restricted for some reason at this stage, however, morphologic abnormality in the posterior polar retina might be incurred.

The purpose of this study, correspondingly, was to compare the distance from the fovea to the Bruch’s membrane opening (BMO) between SIOD eyes and their axial-length-matched normal controls. We also investigated whether the characteristic RNFL profile of SIOD can be normalized after adjusting the circle scan position to the presumptive site based on the mean difference of distance between the SIOD and control groups.

## Results

Fifteen (15) participants (22 eyes) with SIOD were enrolled as the SIOD group, and 44 age and axial-length-matched participants (44 eyes) without SIOD were enrolled as the control group. Fifty-nine (59) participants (66 eyes) were enrolled in this study in total. The subject demographics are summarized in Table 1. There were no significant differences in age or axial length between the SIOD and control groups (P > 0.05).

**Table 1:** Subject demographics.

		SIOD group (N = 22 eyes, 15 subjects)	Control group (N = 44 eyes, 44 subjects)	P-value
Age (years)		59.5 ± 12.6	56.6 ± 15.8	0.525*
Sex	Male	3 (20.00%)	21 (47.73%)	0.059^†^
	Female	12 (80.00%)	23 (52.27%)	
Refractive error (diopters)		− 1.17 ± 0.50	− 1.04 ± 0.33	0.832*
Axial length (mm)		24.27 ± 0.34	24.23 ± 0.19	0.926*

*SIOD* situs inversus of optic disc.

*Comparison was performed using generalized estimating equation (GEE) regression model to account for the paired-eye correlation.

^†^Comparison performed using chi-square test.

**Figure 1:**
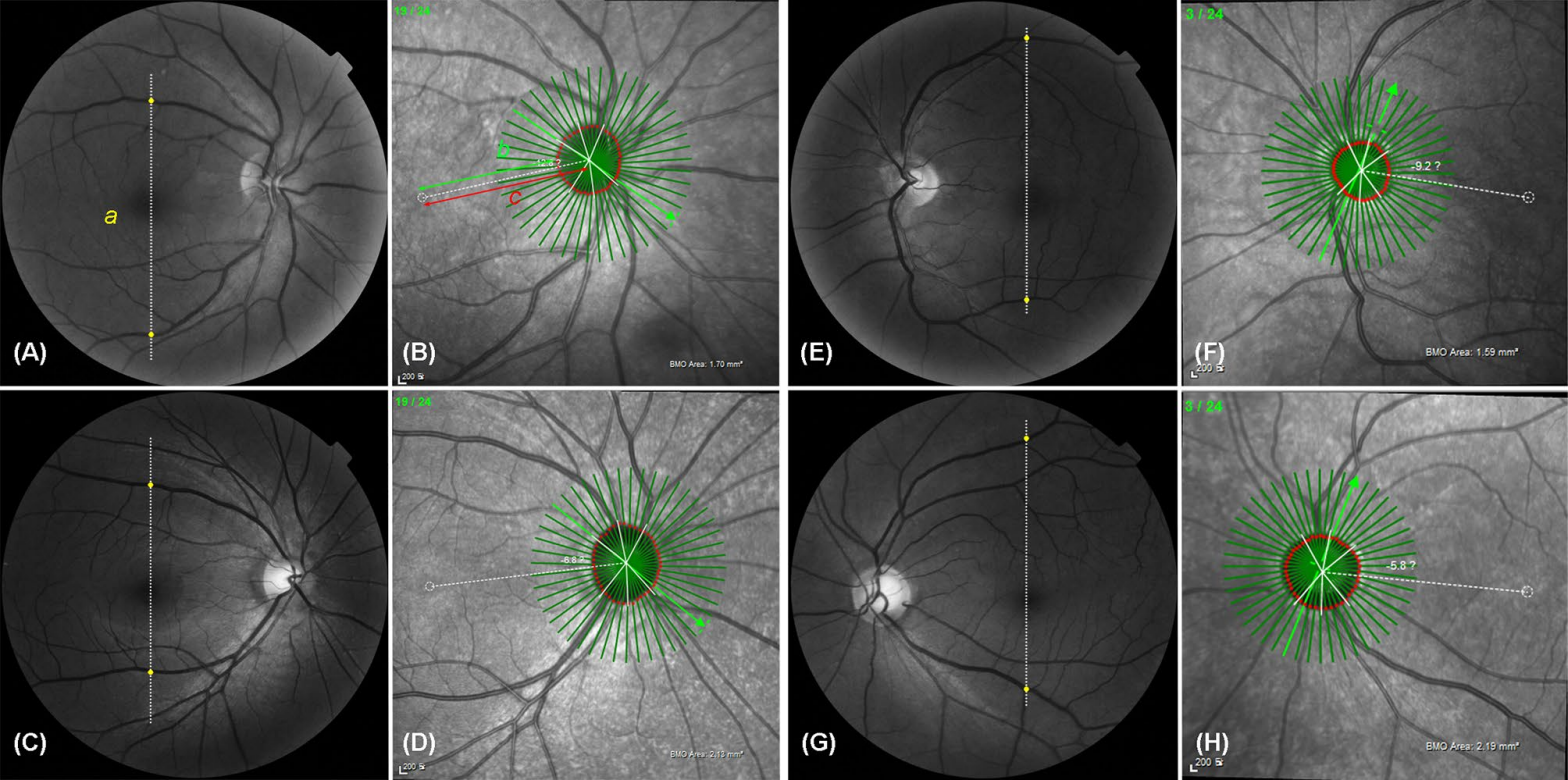
Measurements of fovea-BMO distance and distance between superior and inferior vascular arcades. Red-free fundus photography of SIOD group **(A,E)** and control group **(C,G)**. Infra-red image of SD-OCT of SIOD group **(B,F)** and control group **(D,H)**. The distance between the fovea and BMO was measured in two ways: (1) the distance between the fovea and the BMO center (**c**, foveo-BMO-center distance), and (2) the distance between the fovea and the temporal BMO margin along the fovea-BMO axis (**b**, foveo-BMO margin distance). The vertical distance between the temporal superior and inferior vascular arcades **(a) **was the distance between the crossing points of the vertical line passing the fovea and the superior and inferior venous arcades.

Table 2 shows the comparison of ocular parameters between the SIOD and control groups. The SIOD group had a shorter distance of ‘fovea-BMO center’ and ‘fovea-BMO margin’ relative to the control group (both P < 0.001, Fig. 1).

**Table 2:** Comparison of posterior polar distances between situs inversus of optic disc (SIOD) and control groups.

	SIOD group	Control group	P-value
Foveo-BMO-center distance (μm)	**4215 ± 84**	**4680 ± 39**	** < 0.001**
Foveo-BMO margin distance (μm)	**3404 ± 105**	**3833 ± 42**	** < 0.001**
Foveo-BMO-center axis (°)	– 10.25 ± 1.68	− 7.46 ± 0.50	0.112
Vertical distance between superior and inferior vascular arcades (arbitrary units)	1807 ± 41	1808 ± 41	0.974
BMO area (mm^2^)	2.21 ± 0.18	2.44 ± 0.08	0.248

Comparison was performed using generalized estimating equation (GEE) regression model to account for the paired-eye correlation. Statistically significant values (P < 0.05) appear in boldface.

*BMO* Bruch’s membrane opening.

**Figure 2:**
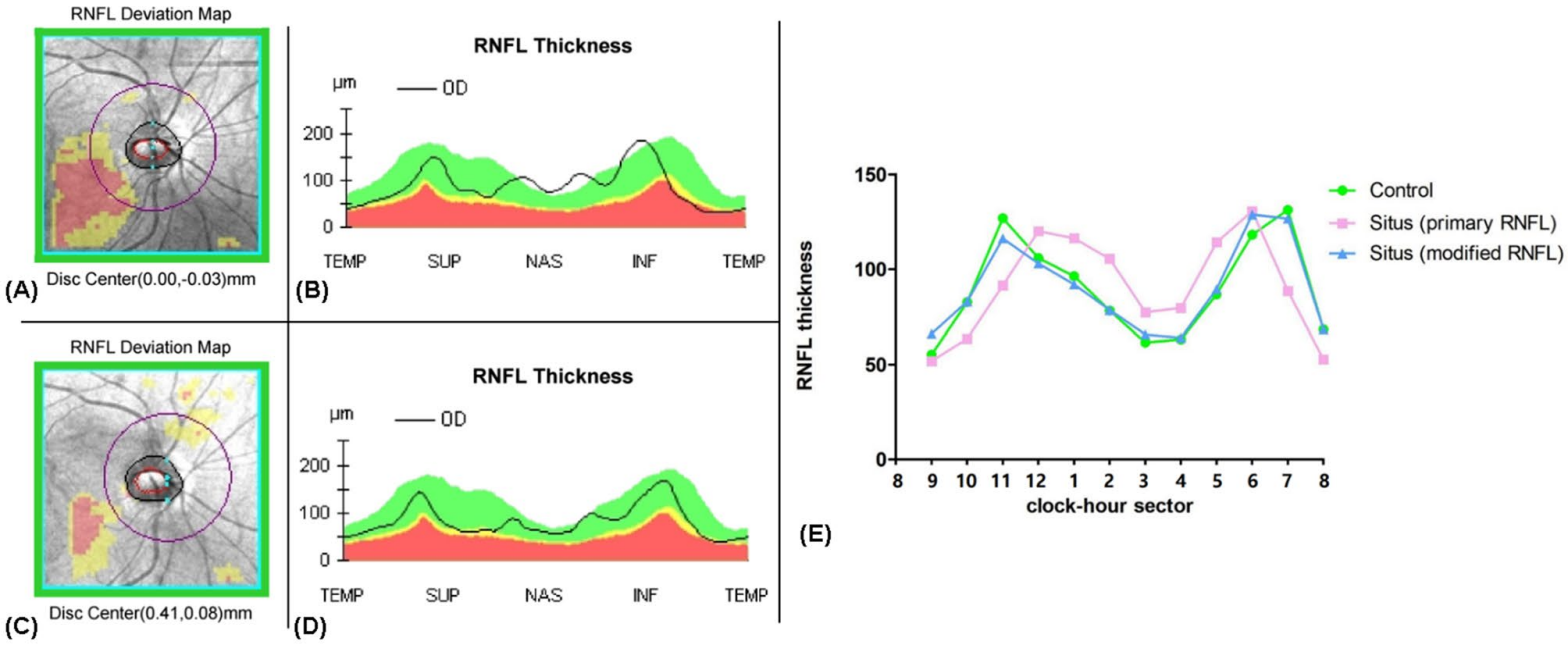
Examples of RNFL deviation map including coordinates of disc center and RNFL thickness map of one SIOD eye. **(A,B) **Initial circumpapillary RNFL thickness profile. **(C,D)** RNFL thickness profile after adjusting circle scan location. **(E)** Circumpapillary RNFL thickness profiles between groups. Please note that the difference between the SIOD and control groups was diminished after adjusting the circle scan location.

The superior and inferior RNFL peak locations in the SIOD group were more nasally located than in the control group (Fig. 2B), which fact corresponds well with a previous study’s finding^3^. We manipulated the optic disc center from the automatically calculated coordinates (Fig. 2A) to coordinates along the foveo-BMO axis at a distance of 465 μm (Fig. 2C). Then, the recalculated RNFL thicknesses of the SIOD group were plotted as a clock-hour thickness map (Fig. 2D), which showed a similar pattern to that of the map of the control group (Fig. 2E).

A comparison between the SIOD and control groups regarding peripapillary RNFL thickness according to clock-hour sectors is provided in Table 3. After Bonferroni correction, a P-value less than 0.0042 (= 0.05/12) was considered statistically significant. The SIOD group had a thicker RNFL in clock-hour sectors 2, 3 and 5 and a thinner RNFL in clock-hour sectors 10, 11 and 7 relative to the control group. The circumpapillary RNFL thickness profile of the SIOD group showed a similar distribution to that of the control group when the circle scan center was adjusted nasally by the amount of the mean difference between the SIOD and control groups (465 μm) along the foveo-BMO axis.

**Table 3:** Comparison of peripapillary retinal nerve fiber layer (RNFL) thickness between situs inversus of optic disc (SIOD) and control groups.

Clock-hour sectors	Condition (least squares mean ± SE)*			P-value	Pairwise comparison		
	Control group	SIOD group	SIOD group after scan circle reposition		Control vs. SIOD	Control vs. repositioned SIOD	SIOD vs. repositioned SIOD
9	55.09 ± 1.80	50.03 ± 3.84	66.88 ± 2.88	** < 0.0001**	0.2330	**0.0005**	** < 0.0001**
10	82.64 ± 2.85	63.84 ± 2.44	79.22 ± 3.89	** < 0.0001**	** < 0.0001**	0.4784	**0.0001**
11	126.84 ± 2.91	90.11 ± 3.96	113.44 ± 5.95	** < 0.0001**	** < 0.0001**	0.0432	** < 0.0001**
12	105.91 ± 3.44	120.43 ± 5.72	100.47 ± 6.87	0.0049	0.0298	0.4794	**0.0026**
1	96.34 ± 2.69	107.94 ± 6.55	86.70 ± 6.18	**0.0003**	0.1014	0.1527	**0.0001**
2	78.23 ± 2.45	103.73 ± 5.52	77.11 ± 4.03	** < 0.0001**	** < 0.0001**	0.8132	** < 0.0001**
3	61.50 ± 1.75	77.31 ± 4.19	66.07 ± 3.40	** < 0.0001**	**0.0005**	0.2318	** < 0.0001**
4	63.07 ± 1.54	80.16 ± 7.42	64.69 ± 4.85	**0.0015**	0.0241	0.7505	**0.0004**
5	86.66 ± 2.47	116.13 ± 6.13	90.47 ± 6.93	** < 0.0001**	** < 0.0001**	0.6045	** < 0.0001**
6	118.16 ± 3.29	130.51 ± 7.80	129.60 ± 9.21	0.3451	0.1447	0.2418	0.8756
7	131.23 ± 3.72	86.53 ± 5.11	126.43 ± 8.45	** < 0.0001**	** < 0.0001**	0.6033	** < 0.0001**
8	68.34 ± 2.11	51.53 ± 2.78	69.10 ± 3.32	** < 0.0001**	** < 0.0001**	0.8459	** < 0.0001**

Statistically significant values after Bonferroni correction (P < 0.0042) appear in boldface.

Comparison was performed using generalized estimating equation (GEE) regression model and pairwise contrast test as post-hoc analyses.

## Discussion

SIOD is characterized by the anomalous direction of retinal vessels: after the emergence of the central retinal vascular trunk, retinal vascular arcades advance nasally initially, followed by an abrupt turn temporally^8^. Similarly to the case of situs inversus visceralis or dextrocardia, SIOD is believed to be caused by congenital malformation. The difference of SIOD from the former, however, is that the reverse appearance is not observed in the left–right asymmetry; rather, the reverse direction of vascular arcade is observed only in the limited range between the optic disc and its nasal vicinity. This study showed that the optic disc was located nearer to the fovea in the SIOD eyes than in the control eyes, but there was no difference in the vertical distance of the major vascular arcade. Further, relocating the optic disc by the normative distance from the fovea normalized the peripapillary RNFL thickness distribution with accordant hump locations. These findings suggest that SIOD is the result of a short foveo-BMO-center distance while the retinal vascular arcades are preserved outside the foveo-BMO range.

On average, axial length is around 17 mm in newborns, and increases rapidly between birth and 2 years of age, gradually reaching to 24 mm by age 10 to 15^5^. This means that every eyeball has to grow after birth regardless of how short it will be in adulthood. In the meantime, eyeball diameter normally increases by a factor of 1.4, and the surface area by a factor of about 2.0^7^. These changes increase the total inner ocular surface to be covered by the retina. Since retinal cytogenesis is already ended before birth, the increased surface area is partially compensated by the expansion of preexisting retinal tissue: either by the increasing volume of cells or by the rearrangement of outer nuclear cell layers^7^. This expansion, however, has a regional difference: the most-peripheral, less differentiated part of the retina becomes stretched much more than the central sensory retina^7^. This is thought to be caused by the regional difference of tissue compliance, which exaggerates further with more stretching^9^. If the retinal compliance of the posterior polar area is lower than usual in certain eyes, the retinal expansion would be disturbed in that limited area, and might produce a shorter distance between the fovea and the optic disc.

We do not know why the foveo-disc distance was shorter in the SIOD group than in the control group without difference in the vertical distance of the major vascular arcade. This result might indicate that posterior polar expansion was restricted only in the transverse direction, not in the vertical direction. The vertically oval shape of the optic disc in newborns contrasts with the rounder BMO shape in adults^10^. During the earlier BMO and retinal expansion period, the more predominant expansion direction in the retina might be along the horizontal meridian. If some eyes have lower retinal compliance of the posterior polar area at this direction, they might have suffered restricted expansion during this phase. Further studies are needed to elucidate the pathophysiology of SIOD in detail.

The peripapillary RNFL thickness profile differed between the SIOD and control groups in this study: the two humps were gathered more on the nasal side^3^. The reverse direction of the myopic peripapillary RNFL thickness profile was shown after measuring with respect to the funduscopic disc center: the two humps were gathered more on the temporal side. Moreover, in myopic eyes, shifting the scan circle to the more temporal side restored the normal thickness profile^11^. Our recent Boramae Myopia Cohort Study answered why such shifting would be reasonable in myopic eyes: the funduscopic temporal margin of the optic disc shifted toward the nasal side of the BMO during myopic axial elongation^12–14^. Likewise, but in the opposite direction, we moved the circle scan to the more nasal side in this study. This also normalized the peripapillary RNFL thickness distribution in the SIOD eyes, while it abnormalized the more myopia-like distribution in the control eyes (Supplemental Table 1).

**Figure 3:**
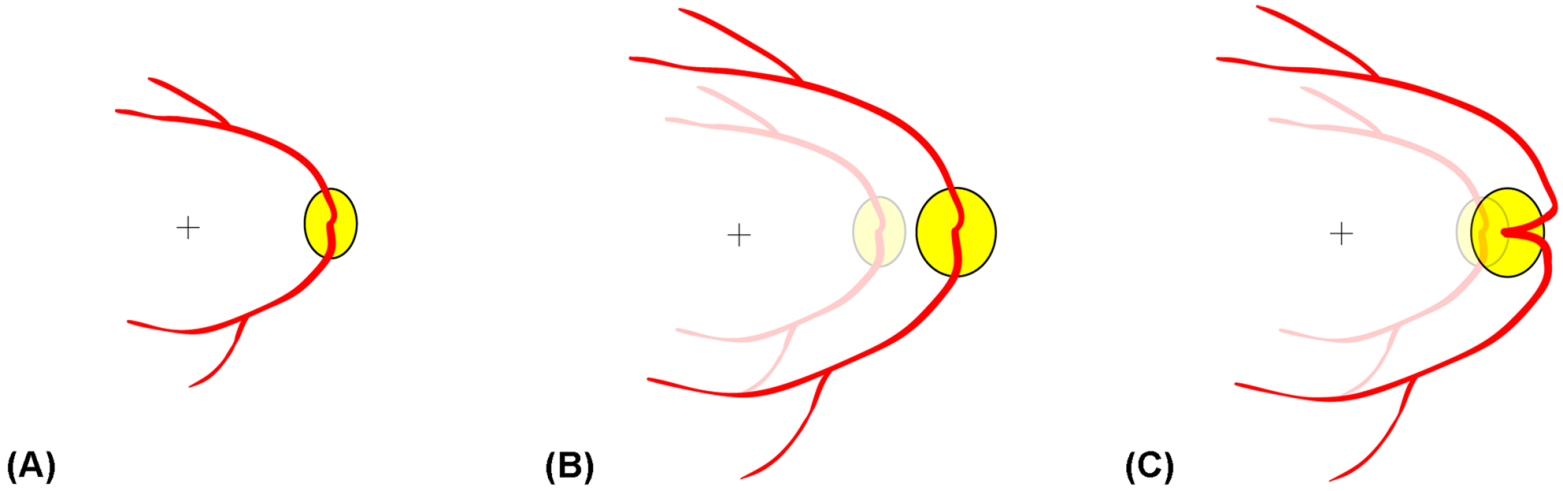
Schematic illustration showing ONH and peripapillary changes during eyeball expansion period. If posterior polar expansion from the neonatal eyeball **(A)** is disturbed only in the transverse direction, it might produce a shorter distance between the fovea and the optic disc **(C) **relative to the case of a normal posterior polar expansion pattern **(B)**.

The axons of retinal ganglion cells are precisely guided through the entire course of their development^15,16^. Such precision is necessary in order to preserve the spatial information received through the retina. Upon its guidance, the axon develops a specialized structure: the growth cone^15,16^. Using this growth cone, the axon grows along the exact road that it should follow by sensing the concentration of attractant or repulsive molecules^15,16^. Such morphogen theory can predict the curves of the RNFL in a simplified mathematical model^17^. Based on these findings, we speculated that the short distance between the optic disc and fovea of SIOD subjects might not be the result of abnormal insertion of the optic nerve to the globe. Instead, we cautiously attributed the reason to the restricted expansion of the posterior polar retina, which occurs after the normal insertion of the optic nerve and the outward growth of retinal ganglion cell axons. If optic nerve insertion close to the fovea were the cause, the RNFL and vascular arcade would follow the normal architecture but in reduced size due to the short distance. According to the morphogen theory, there is no reason for the vascular arcade and the RNFL to have abnormal curves toward the nasal side. The normalization of peripapillary RNFL thickness after moving of the scan circle suggests that normal retinal development might not be interrupted until the stage at which retinal ganglion cells proliferate and finally find the exit of the eyeball: the ONH. Taken together, the reverse direction of retinal vasculature and displaced RNFL profile confined to the optic disc and its nasal vicinity, which phenomena characterize SIOD, suggest that restricted expansion, rather than being predetermined congenitally, might occur after the retinal ganglion cells pass the ONH (Fig. 3). Further longitudinal studies are warranted.

In the Boramae Myopia Cohort Study, we found that the retinal and scleral layers expanded in a different fashion during myopic axial elongation^12–14^. The disproportionate expansion of the outer scleral layer compared with the relatively preserved posterior polar retina and BMO induced the change of border tissue from the internally oblique to the externally oblique configuration^12^, nasal dragging of vascular trunk in the ONH^13^, and the narrow angle between the major vascular arcade and the disc center despite the preserved retinal vascular arcade^13^. If disproportionate expansion of the outer scleral layer in the opposite direction is the cause of SIOD development, the position of the vascular trunk alone should be dragged temporally and the externally oblique configuration should be observed on the nasal ONH border. However, in most cases among the present findings, the vascular trunk position was located centrally, and internally oblique border tissue was observed on the nasal ONH border (data not shown). In the SIOD eyes, no presumptive sign of BMO misalignment from the neural canal opening, such as γ-zone parapapillary atrophy^13,14^, was present; rather, their abnormal peripapillary RNFL profile was settled within the retinal layers including the BMO. Therefore, disproportionate expansion of the outer scleral layer cannot fully explain the development of SIOD.

This study has several limitations. First, our sample size was small because of the rarity of the disease studied. Second, we measured the vertical distance of vascular arcade on red-free fundus photographs in arbitrary units, while we measured the horizontal distance between the fovea and the optic disc on infra-red images of Spectralis OCT since the infra-red images did not cover the whole vascular arcade. To minimize measurement bias, however, we matched two control eyes for every SIOD eye by the axial length. Therefore, we do not believe that this measurement discrepancy affected the results significantly. Lastly, the circumpapillary RNFL thickness measurement should be evaluated with caution. As we showed in this study, the RNFL thickness profile is closely related to the location of the circle scan center, while we do not have any clear reference point for that in Cirrus OCT. Individual factors such as foveo-disc angle might affect the RNFL thickness profile^18^, but we could not adjust them in Cirrus OCT. Further, we arbitrarily repositioned the circle scan in a fixed amount for comparison, which might have induced some bias. The reason we used the RNFL thickness and repositioned the circle scan center, however, was to visualize changes in the general trend of thickness curves, not to demonstrate specific values. For that purpose, the effects of circumpapillary RNFL thickness measurement errors and bias would be negligible.

In conclusion, the SIOD eyes had a shorter foveo-BMO-center distance, and the characteristic peripapillary RNFL thickness profile was normalized by shifting the scan circle more nasally. These findings implicate that, in those eyes, the focal restriction of posterior polar expansion, which occurs after the axons of the retinal ganglion cells find their way out of the eyeball normally, might affect the development of SIOD.

## Methods

The study protocol was approved by the Seoul National University Boramae Medical Center Institutional Review Board and adhered to the tenets of the Declaration of Helsinki.

### Participants

We included SIOD and healthy control subjects from a database of an ongoing prospective cohort study, Boramae Glaucoma Imaging Study (BGIS), at Seoul National University Boramae Medical Center (Seoul, Korea)^19^. Written informed consent was obtained from all participants. All subjects included in this study underwent a full ophthalmologic examination that included best-corrected visual acuity (BCVA) assessment, refraction, slit-lamp biomicroscopy, Goldmann applanation tonometry, gonioscopy, dilated funduscopic examination, keratometry (RKT-7700; Nidek, Hiroshi, Japan), axial length measurement (IOLMaster version 5; Carl Zeiss Meditec, Dublin, California, USA), disc photography and red-free fundus photography (TRC-NW8; Topcon, Tokyo, Japan), and ONH scanning using two spectral-domain optical coherence tomography (SD-OCT) devices. The 200 × 200-cube ONH scans were obtained by SD-OCT (Cirrus HD-OCT; Carl Zeiss Meditec). Twenty-four (24) high-resolution radial scans of the ONH were performed using another SD-OCT device (Spectralis OCT, Heidelberg Engineering, Heidelberg, Germany). During acquisition of both SD-OCT image sets, the subjects were asked to fixate on the target, and images were acquired with the forehead and chin stabilized by the headrest. Extra care was taken during each exam to confirm that the forehead and chin were correctly positioned and did not move.

SIOD, defined as a characteristic emergence of the superior and inferior retinal vessels anomalously nasalward followed by acute arching temporally that is associated with dysversion of the ONH^3,4^, was evaluated by two glaucoma specialists (KML and MK). In cases of disagreement, a final decision was made by a third adjudicator (SHK). Two (2) age- and axial-length-matched healthy control eyes were recruited from the BGIS database for each SIOD eye.

The exclusion criteria were BCVA < 20/40, posterior staphyloma (which can deform the contour of the eyeball) appearing sharply defined in the funduscopic examination, a history of ocular surgery other than cataract extraction or corneal refractive surgery, retinal or neurologic disease, a poor-quality image (i.e., quality score < 15 of Spectralis radial scan images or signal strength < 7/10 of Cirrus cube scan images). If both eyes were eligible, both eyes were used for the analysis in the SIOD group, while in the control group, one eye was randomly selected.

### Measurement of fovea-BMO distance

The peripapillary area was imaged by SD-OCT. The corneal curvature of each eye was entered into the SD-OCT system (Spectralis, Heidelberg Engineering) before performing SD-OCT scanning so as to compensate for potential magnification error. The Glaucoma Module Premium Edition of the Spectralis device enables detection of the BMO. With 24 high-resolution radial scan images of the ONH separated by 15° and each averaged from 24 individual B-scans, SD-OCT automatically detects the margin of the BMO. Every detected BMO margin in the present study was reviewed by one of the authors (YIS), and errors were corrected manually. Based on the edited BMO margin, Spectralis calculated the area and center of the BMO and determined the foveo-BMO axis.

After demarcation of the BMO margin, the distance between the fovea and BMO was measured in the infra-red image of SD-OCT (Fig. 1). The distance from the fovea was measured in two ways: 1) the distance between the fovea and the BMO center (foveo-BMO-center distance), and 2) the distance between the fovea and the temporal BMO margin along the fovea-BMO axis (foveo-BMO margin distance) using Spectralis’ built-in caliper tool.

### Measurement of distance between superior and inferior vascular arcades

The vertical distance between the temporal superior and inferior vascular arcades was measured on red-free fundus photographs^20^. A vertical line was drawn from the fovea, and the crossing points of the superior and inferior venous arcades were marked. The distance between the two crossing points was measured using the built-in caliper tool of MPACS (Medical Picture Archiving and Communication System) with arbitrary units. The venous arcades were chosen for measurement because of the large variations in the arterial arcades.

### Measurement of circumpapillary RNFL thickness

The 200 $\times$ 200-cube ONH scans were performed using SD-OCT (Cirrus HD-OCT, Carl Zeiss Meditec). Cirrus’ built-in algorithm automatically delineates the optic disc margin and calculates the center of the optic disc^21^. Since the thickness profiles were obtained in 200 $\times$ 200 pixels, the center of the circle measuring circumpapillary RNFL thickness could be adjusted after image acquisition^11^. To prove our hypothesis that the abnormal circumpapillary RNFL thickness profile of the SIOD patients was derived from a shorter foveo-BMO-center distance, we intentionally moved the circle scan center nasally by the amount of the mean difference between the SIOD and control groups (465 μm) along the foveo-BMO axis in both groups. The original RNFL thickness profiles and the adjusted RNFL thickness profiles were then compared with those of the control group.

### Statistical analysis

Inter-group comparisons were performed using a generalized estimating equation (GEE) regression model to account for the correlation of paired eyes from the same participants. Due to the internal correlation of the same eye, the circumpapillary RNFL thickness was compared using the GEE with a pairwise contrast test. To overcome multiple comparisons of the 12 locations of RNFL thickness measurement, statistical tests were performed with R statistical packages version 3.4.3 (available at https://www.R-project.org; assessed December 5, 2017). The data herein are presented as the mean ± standard error except where stated otherwise, and the cutoff for statistical significance was set at *P* < 0.05.

## Supplementary information

Supplementary Table 1.

## References

[1] Brodsky MC (1994). Congenital optic disk anomalies. Surv. Ophthalmol..

[2] Caccamise WC (1954). Situs inversus of the optic disc with inferior conus and variable myopia: A case report. Am. J. Ophthalmol..

[3] Kang S, Jin S, Roh KH, Hwang YH (2015). Peripapillary retinal nerve fiber layer and optic nerve head characteristics in eyes with situs inversus of the optic disc. J. Glaucoma.

[4] Kothari M, Chatterjee DN (2010). Unilateral situs inversus of optic disc associated with reduced binocularity and stereoacuity resembling monofixation syndrome. Indian J. Ophthalmol..

[5] Gordon RA, Donzis PB (1985). Refractive development of the human eye. Arch. Ophthalmol..

[6] Patel A (2016). Optic nerve head development in healthy infants and children using handheld spectral-domain optical coherence tomography. Ophthalmology.

[7] Kuhrt H (2012). Postnatal mammalian retinal development: Quantitative data and general rules. Prog. Retinal Eye Res..

[8] Baneke AJ, Williams KM, Mahroo OA, Mohamed M, Hammond CJ (2018). A twin study of cilioretinal arteries, tilted discs and situs inversus. Graefes Arch. Clin. Exp. Ophthalmol..

[9] Reichenbach, A. *et al.* Development of the rabbit retina. IV. Tissue tensility and elasticity in dependence on topographic specializations. *Exp. Eye Res.* **53**, 241–251 (1991). 10.1016/0014-4835(91)90080-x 1915681

[10] Kim M, Kim SY, Lee KM, Oh S, Kim SH (2019). Position of central vascular trunk and shape of optic nerve head in newborns. Invest. Ophthalmol. Vis. Sci..

[11] Chung JK, Yoo YC (2011). Correct calculation circle location of optical coherence tomography in measuring retinal nerve fiber layer thickness in eyes with myopic tilted discs. Invest. Ophthalmol. Vis. Sci..

[12] Kim M, Choung HK, Lee KM, Oh S, Kim SH (2018). Longitudinal changes of optic nerve head and peripapillary structure during childhood myopia progression on OCT: Boramae myopia cohort study report 1. Ophthalmology.

[13] Lee KM, Choung HK, Kim M, Oh S, Kim SH (2018). Positional change of optic nerve head vasculature during axial elongation as evidence of lamina cribrosa shifting: Boramae myopia cohort study report 2. Ophthalmology.

[14] Lee KM, Choung HK, Kim M, Oh S, Kim SH (2018). Change of beta-zone parapapillary atrophy during axial elongation: Boramae myopia cohort study report 3. Invest. Ophthalmol. Vis. Sci..

[15] Oster SF, Deiner M, Birgbauer E, Sretavan DW (2004). Ganglion cell axon pathfinding in the retina and optic nerve. Semin. Cell Dev. Biol..

[16] Erskine L, Herrera E (2007). The retinal ganglion cell axon's journey: Insights into molecular mechanisms of axon guidance. Dev. Biol..

[17] Airaksinen PJ, Doro S, Veijola J (2008). Conformal geometry of the retinal nerve fiber layer. Proc. Natl. Acad. Sci. U S A.

[18] Akbari M, Nikdel M, Moghimi S, Subramanian PS, Fard MA (2019). Effect of foveal location on retinal nerve fiber layer thickness profile in superior oblique palsy eyes. J. Glaucoma.

[19] Lee KM, Kim M, Oh S, Kim SH (2018). Position of central retinal vascular trunk and preferential location of glaucomatous damage in myopic normal-tension glaucoma. Ophthalmol. Glaucoma.

[20] Wilson C, Theodorou M, Cocker KD, Fielder AR (2006). The temporal retinal vessel angle and infants born preterm. Br. J. Ophthalmol..

[21] Shin JW (2016). The effect of optic disc center displacement on retinal nerve fiber layer measurement determined by spectral domain optical coherence tomography. PLoS ONE.

