# Supplementary material for: Short foveo-disc distance in situs inversus of optic disc
Source: Sci Rep. 2020 Oct 20;10:17740. doi: 10.1038/s41598-020-74743-0 (PMC7576120; doi:10.1038/s41598-020-74743-0)
Supplement: Supplementary file 1 — Supplementary Table 1. [file 41598_2020_74743_MOESM1_ESM.docx]

**Short foveo-disc distance in situs inversus of optic disc**

**Running Head:** Short foveo-disc distance in SIOD

Young In Shin.^1,2*^, Kyoung Min Lee ^1,3*^, Martha Kim^4^, Sohee Oh^3^, Seok Hwan Kim ^1,3^

^1^ Department of Ophthalmology, Seoul National University, College of Medicine, Seoul, Korea

^2^ Department of Ophthalmology, Seoul National University Hospital, Seoul, Korea

^3^ Department of Ophthalmology, Seoul National University Boramae Medical Center, Seoul, Korea

^4^ Department of Ophthalmology, Dongguk University Ilsan Hospital, Goyang, Korea

*Correspondence to:* Seok Hwan Kim

Department of Ophthalmology, Seoul National University Boramae Medical Center, 39 Boramae Road, Dongjak-gu, 07061, Seoul, Korea

Tel: 82-02-870-2415, Fax: 82-02-831-2826, e-mail: xcski@hanmail.net

^*^Young In Shin and Kyoung Min Lee equally contributed to the work and therefore should be considered as equivalent authors.

**Supplemental Table 1. Comparison of peripapillary retinal nerve fiber layer (RNFL) thickness between situs inversus of optic disc (SIOD) and control groups including scan circle reposition in control group**

| **Clock-hour sectors** | **Condition (least squares mean ±SE)*** | | | | **P-value** | **Pairwise comparison** | | | | |
| --- | --- | --- | --- | --- | --- | --- | --- | --- | --- | --- |
|  | **Control group** | **SIOD group** | **SIOD group after scan circle reposition** | **Control group after scan circle reposition** |  | **Control vs. SIOD** | **Control vs. repositioned SIOD** | **SIOD vs. repositioned SIOD** | **Control vs. repositioned Control** | **SIOD vs. repositioned Control** |
| 9 | 55.09±1.80 | 50.25±3.88 | 67.11±2.91 | 66.68±1.71 | **<0.0001** | 0.2580 | **0.0004** | **<0.0001** | **<0.0001** | **0.0001** |
| 10 | 82.64±2.85 | 64.38±2.22 | 79.76±3.59 | 101.09±3.48 | **<0.0001** | **<0.0001** | 0.5308 | **0.0001** | **<0.0001** | **<0.0001** |
| 11 | 126.84±2.91 | 90.12±3.95 | 113.45±5.94 | 133.18±2.92 | **<0.0001** | **<0.0001** | 0.0430 | **<0.0001** | **0.0021** | **<0.0001** |
| 12 | 105.91±3.44 | 120.42±5.72 | 100.47±6.85 | 92.36±2.77 | **<0.0001** | 0.0297 | 0.4781 | **0.0026** | **<0.0001** | **<0.0001** |
| 1 | 96.34±2.69 | 108.05±6.62 | 86.81±6.24 | 85.25±2.34 | **<0.0001** | 0.1015 | 0.1608 | **0.0001** | **<0.0001** | **0.0012** |
| 2 | 78.23±2.45 | 103.72±5.52 | 77.10±4.03 | 65.98±1.93 | **<0.0001** | **<0.0001** | 0.8112 | **<0.0001** | **<0.0001** | **<0.0001** |
| 3 | 61.50±1.75 | 77.32±4.24 | 66.08±3.47 | 55.75±1.67 | **<0.0001** | **0.0006** | 0.2383 | **<0.0001** | **<0.0001** | **<0.0001** |
| 4 | 63.07±1.54 | 80.15±7.34 | 64.68±4.44 | 53.48±1.40 | **<0.0001** | 0.0228 | 0.7480 | **0.0004** | **<0.0001** | **0.0004** |
| 5 | 86.66±2.47 | 116.16±6.12 | 90.49±6.91 | 72.00±2.02 | **<0.0001** | **<0.0001** | 0.6014 | **<0.0001** | **<0.0001** | **<0.0001** |
| 6 | 118.16±3.29 | 130.71±7.79 | 129.81±9.23 | 100.57±3.20 | **<0.0001** | 0.1379 | 0.2343 | 0.8756 | **<0.0001** | **0.0003** |
| 7 | 131.23±3.72 | 86.20±5.21 | 126.11±8.50 | 141.07±3.74 | **<0.0001** | **<0.0001** | 0.5813 | **<0.0001** | **<0.0001** | **<0.0001** |
| 8 | 68.34±2.11 | 51.76±2.87 | 69.33±3.41 | 90.07±2.85 | **<0.0001** | **<0.0001** | 0.8058 | **<0.0001** | **<0.0001** | **<0.0001** |

Comparison was performed using generalized estimating equation (GEE) regression model and pairwise contrast test as post-hoc analyses.

Statistically significant values after Bonferroni correction (P<0.05/12=0.0042) appear in boldface.
